# Evaluation of the TrueBeam machine‐performance‐check (MPC): Collimator device check (CDC)

**DOI:** 10.1002/acm2.70171

**Published:** 2025-07-14

**Authors:** Michael Barnes, Andrew Dipuglia, Brad Beeksma, Joerg Lehmann

**Affiliations:** ^1^ Department of Radiation Oncology Calvary Mater Hospital Newcastle Newcastle New South Wales Australia; ^2^ School of Informational and Physical Sciences University of Newcastle Newcastle New South Wales Australia; ^3^ Institute of Medical Physics University of Sydney Sydney New South Wales Australia

**Keywords:** MLC, MPC, Picket Fence, QA

## Abstract

**Purpose:**

To evaluate the Varian machine performance check (MPC) collimator devices check (CDC) for routine MLC and jaw testing as part of an AAPM compliant linac QA program.

**Methods:**

CDC MLC positioning, MLC backlash, jaw positioning, and jaw parallelism were each assessed for repeatability and concordance with conventional QA. MLC and jaw positioning were also assessed for sensitivity. Measurement time and repeatability of CDC were assessed by timing and recording five successive measurements on a single linac. Concordance was assessed monthly over 5 months on four linacs, conducted during the same session as conventional QA. MLC positioning was compared to an advanced picket fence test, while jaw positioning and parallelism were compared to department in‐house EPID based methods. MLC backlash was compared to the Varian built‐in method. Sensitivity was assessed via deliberately introduced errors except for MLC backlash, which was assessed via correlation between methods across leaf banks.

**Results:**

CDC requires 4:09 (min:s) ± 1.8 s (2 SD) to perform. Repeatability was measured to be: 0.02 mm for both MLC positioning and backlash, 0.15 mm for jaw positioning and 0.009° for jaw parallelism (2 SD). Concordance was observed for mean MLC positioning to within 0.32 , 0.08 mm for MLC backlash, 0.6 mm for jaw positioning and 0.06° for jaw parallelism. MLC and jaw positioning sensitivity were observed with maximum mean difference between methods of 0.18  and 0.71 mm, respectively. MLC backlash correlation coefficient between methods across leaf banks was observed to 0.84 and 0.9 for banks A and B, respectively.

**Conclusion:**

MPC CDC has been demonstrated to provide acceptably equivalent MLC and jaw positioning assessment to standard methods and could conceivably be used in a linac QA program for these purposes.

## 1 INTRODUCTION

Varian (Varian Medical Systems, Palo Alto, California, USA) released the machine performance check (MPC) application as a standard inclusion on their TrueBeam linear accelerator (linac) with software version 2.0. Subsequently, Varian released it also on the Halcyon linac. MPC is fully integrated with the linac and utilizes automated kilovoltage (kV) and Megavoltage (MV) beam image acquisitions both with and without a custom phantom in place to assess the performance of linac critical parameters on a daily basis prior to clinical use. On the Halcyon system, it is mandatory for MPC to be performed and all associated tests to pass prior to commencement of treatment that day.

Initially, there were two categories of MPC tests; Beam constancy and Geometric, which have been evaluated in the literature for use in linac QA.^1^, ^2^, ^3^, ^4^, ^5^, ^6^, ^7^, ^8^, ^9^, ^10^, ^11^, ^12^ Varian have subsequently updated MPC with additional functionality such as the x‐ray tube alignment procedure evaluated by Barnes et al.^13^ and now also with an additional test suite named the collimator device check (CDC). The CDC was originally released as part of the linac Customer Acceptance procedure, available only to the Varian installation engineers. However, with the release of TrueBeam V3.0, the CDC has now been made available to the customer as a standard MPC module.

The MPC CDC utilizes MV Electronic‐Portal‐Imaging‐Device (EPID) images, acquired without the MPC phantom in place to capture a series of test patterns defined by both multi‐leaf collimator (MLC) and jaws. This approach provides a more thorough examination of the MLC and jaw performance compared to the standard MPC Geometric checks. In particular, jaw and MLC positional accuracy are assessed at multiple points along their travel rather than at single points as per standard MPC.

With regard to the MLC, the multiple‐point quantitative positional accuracy assessment is analogous to a quantitative Picket fence test. If the accuracy of this assessment can be demonstrated, the CDC could potentially fulfill the recommendations of AAPM for routine quantitative MLC positional accuracy testing.^14^, ^15^, ^16^ MPC CDC is particularly relevant to the most recent AAPM linac QA guidelines, Medical Physics Practice Guideline (MPPG) 8.b, which recommends; a quantitative assessment of MLC position on a weekly basis, a tightened action limit of ± 0.5 mm for MLC positional accuracy and more generally that “the frequency and rigor of MLC QA tests must increase.” ^16^ It is hypothesized that the MPC CDC checks may be able to meet these recommendations better than many currently used MLC tests. Given the MPC CDC is both accessible and straightforward to perform on all Varian TrueBeam linacs it may provide a practical solution for departments, particularly those with limited resources, to ensure their MLC static positioning meet the required standards. It is the aim of this study to evaluate the equivalency of CDC with respect to conventional QA methods, with the aim of assessing its utility for integration into routine linac QA programs.

## 2 METHODS

### 2.1 Materials

#### 2.1.1 MPC collimator check device (CDC) tests

The CDC utilizes a sequence of jaw and MLC defined MV EPID images. Initially, an MLC comb pattern is imaged at five different collimator angles, from which the center of collimator rotation is determined. This is used as the spatial reference point for all subsequent tests. The CDC then utilizes further test plan images to evaluate MLC positional accuracy and reproducibility as well as positional accuracy and parallelism of the jaws. All imaging is performed using the 6 MV beam. The methodology underlying the CDC is outlined in Appendix B of the Varian Installation Product Acceptance (IPA),^17^ and summarized in the following.

##### MPC CDC MLC positional accuracy and reproducibility/backlash

**FIGURE 1:**
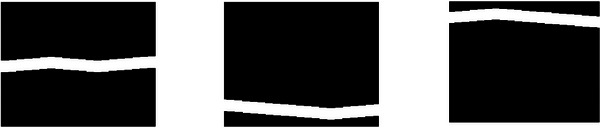
Representations of the three MLC test patterns used in CDC. Left = Pos1, center = Pos2 and Right = Pos3. Adapted from Varian IPA‐HT‐3X‐C.^17^

To assess MLC positional accuracy, three distinct MLC test patterns are imaged. Each pattern consists of a 2 cm MLC gap arranged in a wave‐like pattern, where groups of three adjacent leaf pairs are offset by 2 mm relative to one another, as illustrated in Figure 1. The three patterns encompass the largest possible range of MLC travel, thereby incorporating MLC carriage shifts. For each pattern, the position of each MLC leaf is identified on the image and the distance from the measured collimator rotation axis is recorded. This distance is then compared to expected position, with discrepancies evaluated against a 1 mm non‐customizable threshold. Each test pattern is imaged twice, with the MLC leaves first moved away from their nominal position before returning to it for the second image. Reproducibility is assessed as the absolute positional difference between the two iterations, with a 0.5 mm threshold applied. This serves as a measure of backlash in the MLC leaf drive mechanism. From the vendor documentation it is unclear to the user which image, or whether an average is utilized for the positional test. Notably, when the MPC MLC reproducibility test results are exported to .csv, they are labelled as “backlash”; therefore, the term “backlash test” will be used henceforth to refer to this assessment.

##### MPC CDC Jaw Positioning Accuracy and Parallelism

MPC CDC evaluates jaw positioning accuracy at four distinct points per jaw, spanning a total of 19 cm jaw travel. This is achieved with eight jaw‐defined fields (four for X jaws and four for Y jaws) with jaw positions set at 1, 0, ‐10 and ‐18 cm for the X1 and Y1 jaws and 18, 10, 0, ‐1 cm for the X2 and Y2 jaws as per International Electrotechnical Commission, IEC1217 scale convention.^17^ In each image, the positions of the opposing jaws are detected at the center of the jaw face with jaw position recorded as the point on the profile perpendicular to the jaw face, which has the steepest gradient. The distance from this point to the measured collimator axis is recorded. This distance is then compared to the expected distance, with a threshold of 1 mm applied for the X jaws and 2 mm for the Y jaws. The parallelism of each jaw relative to the MLC is assessed by measuring the angular deviation from parallel for the X jaws, and the angle of orthogonality for the Y jaw. A threshold of 0.4° is applied for both measurements. While the assessment of the Y jaw is technically a measure of orthogonality, this test will henceforth generically be referred to as “parallelism.”

#### 2.1.2 Conventional QA

The local department monthly quantitative MLC positional accuracy test is the Stakitt test, which is a modified version of the original method of Bayouth et al.^18^ The Stakitt methodology is presented in Barnes et al.^19^ and Barnes et al.^20^ In short, the method utilizes EPID imaging of an MLC test field composed of a series of 6 × 2 cm^2^ strip fields including one carriage shift in between strips 3 and 4. In the image, individual MLC leaf positions are detected for both MLC banks defined as the point on the profile perpendicular to the leaf face, which has the steepest gradient. The distance per leaf from the collimator rotation axis measured on the day is recorded. The method shares many aspects with MPC CDC, but additionally includes functionality for removing the influence of EPID panel skew and collimator rotation readout inaccuracy. If these are not accounted for then a rotation between the MLC and EPID can occur that will result in a small measurement inaccuracy.

The local departmental method for assessing jaw positional accuracy is also EPID‐based and follows the asymmetric jaw position test approach outlined by Clews and Greer.^21^ In this methodology, the positions of individual jaws at their zero positions are compared between opposing jaws to assure accurate junctions. Individual jaw positions are measured relative to the collimator axis in a manner similar to the MPC CDC technique, allowing for direct comparison between the two methods. The methodology is also applied at the 10 cm off‐axis positions per jaw.

Jaw parallelism is not directly evaluated in the local department's QA program. However, the program includes EPID‐based measurements of both jaw and MLC‐defined fields, which were re‐analyzed to assess jaw parallelism. These results were then compared to those obtained from the MPC CDC results.

#### 2.1.3 Data acquisition and analysis software

Data for this study was acquired on two TrueBeam linacs with Millennium MLC and two TrueBeam Edge linacs with HD MLC. All machines utilize the aS1200 EPID panel at 100 cm source‐to‐detector‐distance (SDD). Local department tests are all analyzed using customized V2022b Matlab scripts (Mathworks Inc, Natick, Massachusetts, USA). Comparison between MPC and local results was performed using Microsoft Excel 2016 (Microsoft Corporation, Redmond, Washington, USA).

### 2.2 Measurement methods

#### 2.2.1 Time study and repeatability

To assess the repeatability of the MPC CDC tests and to assess the time required to perform CDC, five consecutive CDC measurements were conducted on a single TrueBeam with Millennium MLC. A stopwatch was used to measure the acquisition time for the CDC, from mode up of the CDC test to the availability of results. The repeatability of each test was quantified as two standard deviations about the mean.

#### 2.2.2 Concordance with conventional QA methods

The MPC CDC was performed each month in the same measurement session as the local department EPID‐based monthly QA including MLC (Stakitt) and jaw positioning tests on four TrueBeams (two Millennium MLC and two HD MLC) for a period of 5 months. In this period, five data points each were acquired on three of the linacs and six data points were acquired on the fourth linac.

##### MLC positional accuracy

The measured MLC positions from MPC CDC were compared against the corresponding positions recorded by Stakitt at similar points along the leaf trajectory. The CDC positions “Pos 2” and “Pos 3” did not align with any corresponding leaf positions in the Stakitt test data, so the CDC position “Pos 1” was compared against Stakitt strip 3 for the Bank A leaves and strip 4 for the Bank B leaves as the closest comparable points. These Stakitt strip points are within 1 cm of the average corresponding CDC points. It is important to note that a carriage shift occurs between strip number 3 and 4 of the Stakitt delivery.

##### MLC backlash

To evaluate MPC CDC assessment of MLC backlash via the MLC reproducibility test, one instance of running MPC CDC on the same day as the Varian built‐in backlash test was performed when the backlash test was due as part of the linac's regular preventative maintenance schedule. The built‐in Varian backlash test methodology is described in Barnes et al.^19^ and in summary involves moving the MLC leaf into position to break the infrared beam and then retracting the leaf and counting the number of motor counts, converted to a distance, required before the leaf clears the beam. For this study, three iterations were performed and results were averaged. Differences in results were assessed per MLC leaf to the MPC CDC backlash results in the context of their measured repeatability. Additionally, correlation was also assessed across each of the leaves for both MLC banks.

##### Jaw positional accuracy

The jaw positions measured by the MPC CDC were compared against those obtained using the local department method at corresponding points along the jaw trajectory. Assessment was made at both 0 cm off‐axis and 10 cm off‐axis distance for which both MPC CDC and the local method both share the same measurement points.

##### Jaw parallelism accuracy

The department routine QA program includes acquisition of EPID images of both a 10 × 10 cm^2^ jaw‐defined field and a 10 × 10 cm^2^ MLC‐defined field, both with the collimator angle set to 90°. Jaw parallelism was calculated from these images and compared against MPC CDC. For each EPID image, vectors representing the edges of the MLC or jaw are determined. In the case of the MLC‐defined field, vectors of the MLC edges defining the field in the non‐leaf end direction are identified. The skewness angle of the MLC relative to the MV panel coordinate system is then calculated from these edge vectors and averaged, providing a measure of the MLC's skew relative to the MV panel. Similarly, for the jaw‐defined field, vectors of the jaw edges corresponding to the X1, X2, Y1, and Y2 jaws are extracted from the EPID image. The angle from parallel/orthogonal of each jaw relative to the MV panel coordinate system is determined from these vectors. The relative parallelism angle of each jaw with respect to the MLC is then calculated using the MV panel coordinate system as the reference. Given that the MV panel coordinate system remains static between the acquisitions of the jaw‐defined and MLC‐defined fields, it serves as a stable reference and does not introduce additional uncertainties into the evaluation. Furthermore, the collimator angle is also kept constant between field deliveries, thus eliminating potential uncertainties related to collimator angle reproducibility.

CDC assesses the jaw parallelism at multiple positions over a 19 cm span. To validate the accuracy of the CDC assessment of jaw parallelism, the parallelism of each jaw relative to the MLC obtained through the CDC method was compared with the values obtained by the department method for corresponding jaw positions. The evaluation was assessed in the context of the Varian Customer Acceptance threshold for MPC parallelism of 0.4°.^17^

#### 2.2.3 Jaw positioning sensitivity

To evaluate the sensitivity of the CDC jaw positioning tests, a deliberate offset in the range of 1–2 mm was introduced to each of the four jaws, based on standard jaw calibration procedures using graph paper.^22^ In this process, instead of aligning the jaws correctly on the graph paper at the two calibration points the jaws were set with attempted 2 mm deliberate systematic offsets. Due to the relatively large uncertainties associated with the graph paper method the magnitude of the actual introduced offsets is also uncertain. It was estimated that an approximately 2 mm offset was actually introduced into each of the jaws except the Y1 jaw for which the magnitude was likely closer to 1 mm. Pre‐ and post‐adjustment measurements were performed consecutively using the MPC CDC and the departments in‐house EPID‐based jaw positioning tests. The recorded positional changes were compared at the 0 cm points, which are consistent across the different methods along the jaw trajectory. Additionally, the jaw backlash test built‐in to the TrueBeam was also performed for all four jaws to further assess performance. Documentation as to how the jaw backlash test is performed is not available to the customer. Viewing the test visually the jaw is first moved to a point in the negative direction (‐2 cm for x jaws and ‐10 cm for y jaws) and then moved in the positive direction (+20 cm for x jaws and + 10 cm for y jaws). A single backlash result is presented, which from the results graph presented appears to be measured at the change in direction point. It is thus surmised that the test is a push/pull type test likely measuring the motor counts required to change the jaw position as per the encoder readout after the direction change in motion. If so, then this method is similar to how the MLC backlash test operates. The test was set to run three times and an average result recorded.

#### 2.2.4 MLC positioning sensitivity

The CDC MLC positioning sensitivity was assessed via two experiments performed during the decommissioning of a TrueBeam STx (HD MLC) and install of a TrueBeam Edge (HD MLC), respectively.

##### MLC gap adjustment experiment

The Stakitt test at gantry zero degrees (G0) was performed together with MPC CDC measurements. In TrueBeam service mode, the MLC gap parameter was increased by 0.5 mm, resulting in an expected MLC positional change of 0.25 mm in each leaf of both banks. Following this adjustment, both MPC methods and Stakitt were re‐acquired. This process was repeated for additional increases of 0.5  and 1.0 mm in the MLC gap, generating an initial reference data set along with subsequent datasets for 0.25 , 0.5, and 1.0 mm nominal leaf positional errors. These introduced errors equate to MLC positional errors of magnitude within tolerance, at the MPPG 8.b. action limit and then at the TG‐142 tolerance and MPC threshold, respectively. Given that altering the MLC gap parameter uniformly shifts all leaves within a bank, the mean positional change across all leaves within each bank was assessed. The spread in results across leaves was quantified using two standard deviations of the individual leaf results. To assess the measured change in each leaf's position the initial (no error) measured mean position was subtracted from each of the introduced error measurements to provide a measured change in mean leaf position for each method. The difference was then calculated between MPC CDC and Stakitt. This analysis approach was chosen because the measured change in position is the critical factor for sensitivity testing. As there is no ground truth in this experiment, the comparison between MPC methods and the departments standard MLC test (Stakitt) serves as the primary means of assessing equivalency.

##### MLC offset adjustment experiment

This experiment was conducted similarly to the MLC gap experiment, with the primary difference being the adjustment of the MLC offset parameter rather than the gap parameter. While the gap parameter modifies each bank in opposite directions, the offset parameter shifts both MLC banks in the same direction. During machine install the gap offset parameter is optimized. Prior to adjustment the Stakitt test at G0 was performed alongside the MPC CDC. Based on the results, a 0.2 mm adjustment was made to the MLC offset, shifting Bank B toward Bank A. Following adjustment, both the MPC CDC and Stakitt at G0 were repeated. The mean change in MLC positions were then calculated for both MLC banks. The results from MPC CDC and Stakitt were subsequently compared to assess the agreement between the two measurement methods. The measured mean change in MLC positions were then calculated for both banks and both MPC CDC and Stakitt with the results between the two measurement methods compared.

### 2.3 Data analysis

To compare the MPC CDC to the local standard QA method the results data were analyzed as follows: For the concordance and sensitivity tests (MLC position, jaw position, and jaw parallelism), mean and maximum difference values were recorded for each method and compared. The differences were assessed in the clinical context of the associated test tolerance. The spread in results for each method was reported in each case as two standard deviations about the mean. For the MLC experiments, the mean and standard deviations were calculated from individual leaf results across the entire MLC bank, whereas for the jaw positioning and parallelism experiments, the mean and standard deviations were calculated per jaw across the longitudinal data inclusive of all linacs. For the MLC backlash, test results were calculated as the mean result per leaf with the two methods plotted together. Correlation across the leaf banks between backlash methods was also calculated using the Pearson method.

## 3 RESULTS

### 3.1 Time study and repeatability

The average time required to perform CDC was measured at 4:09 min:sec ± 1.8 s (2 SD). This includes setup, data acquisition, analysis and reporting. The repeatability of the MLC mean offset, which includes all MLC leaves at each of the three positions, was measured to be 0.02 mm (2SD) for both banks A and B. The repeatability of the jaw max parallelism was worst of the four jaws for the Y2 jaw measured to be 0.009 ° (2SD). The worst repeatability for the jaw max parameter, which takes the maximum deviation across all of the measured positions per jaw was 0.15 mm (2SD) for the Y1 jaw. For the MLC backlash data the average repeatability across the leaves was measured to be 0.02 mm (2SD) for both MLC banks A and B.

### 3.2 Concordance with conventional QA methods

#### 3.2.1 MLC positional accuracy

**FIGURE 2:**
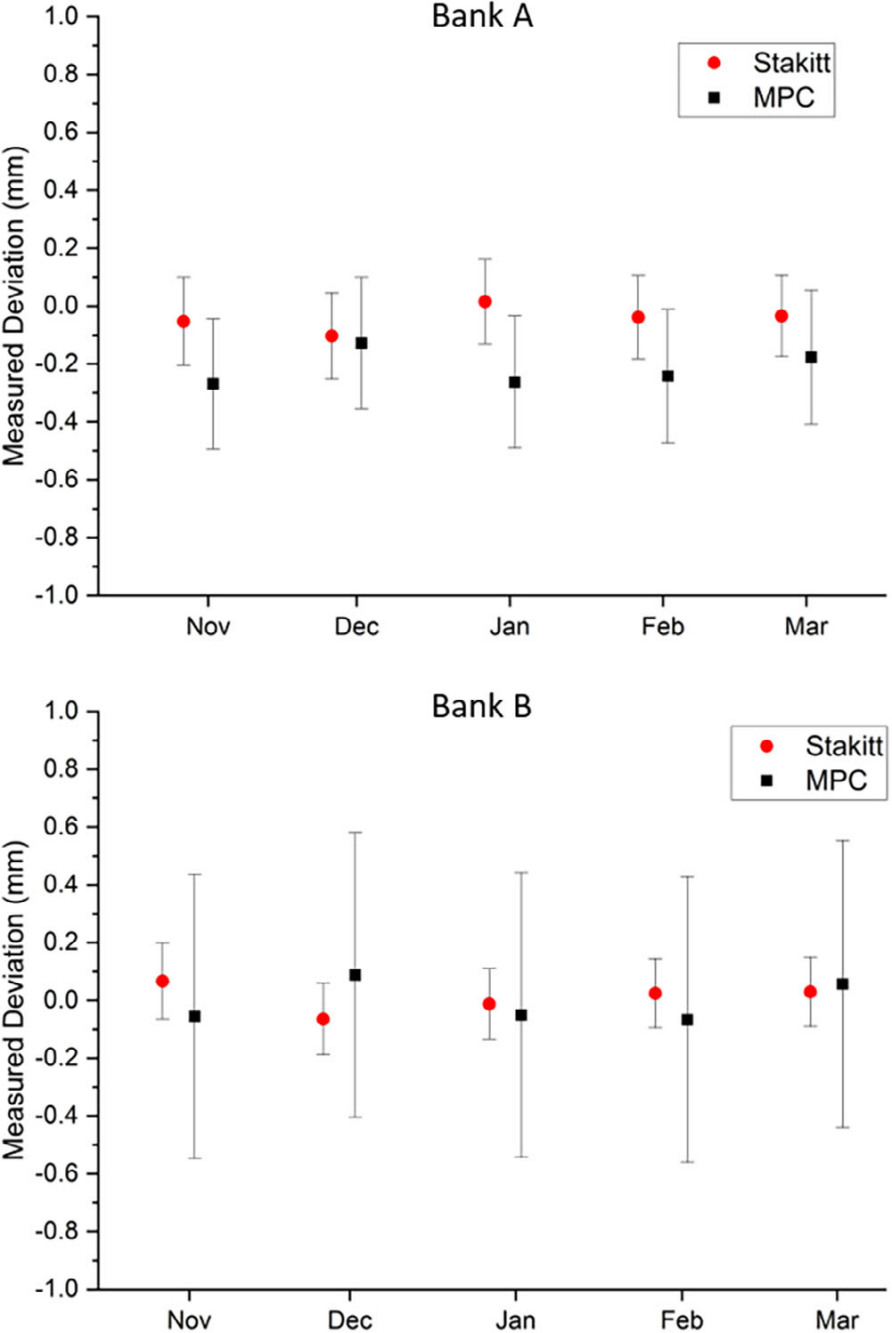
Mean MPC CDC measured MLC positional accuracy compared to the Stakitt test for the worse case linac over a five month period (*n* = 5). Each month CDC and Stakitt data was collected on the same day, but are presented slightly offset along the x‐axis in the figure for readability. Error bars represent ± 2 SD across individual leaves. The y‐axis scale represents the MPC defined threshold range for this test.

Mean MPC CDC MLC positional results alongside the mean Stakitt results over the 5‐month period for one linac are presented in Figure 2.

From the results of Figure 2, the Bank B mean agreement between CDC and Stakitt oscillates about 0 mm. In contrast there is a systematic difference in Bank A mean agreement of up to 0.32 mm, with MPC systematically reporting a larger magnitude measured deviation than Stakitt. The maximum difference observed for an individual leaf was 0.61 mm. It is noteworthy that the data presented in Figure 2 represents the linac with the worst observed agreement. The spread in MPC results across Bank B, is consistently recorded at approximately ± 0.49 mm (2SD) and is approximately double that observed in any of the other three linacs investigated. It is unknown why this is the case. Across all linacs, the mean deviation was ‐0.13 mm for Bank A and 0.11 mm for Bank B.

#### 3.2.2 MLC backlash

**FIGURE 3:**
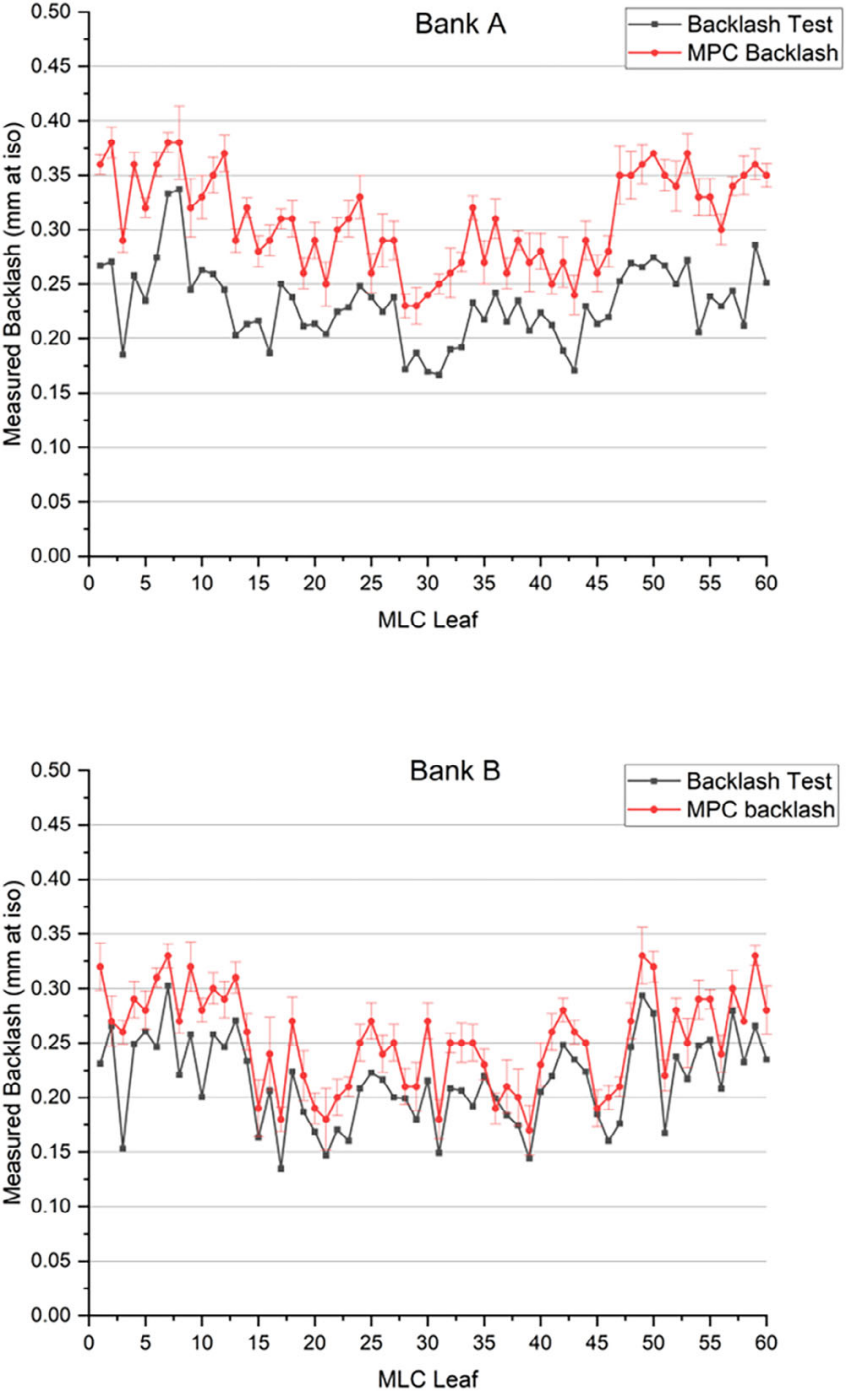
Measured MLC backlash per MLC leaf as measured by both the built‐in backlash test and MPC CDC backlash test for Bank A (top) and Bank B (bottom). Error bars represent two standard deviations from the MPC repeatability data (*n* = 5) per leaf. Error bars are small to be indistinguishable for the built‐in backlash test. The y‐axis scale represents the MPC defined threshold range for this test.

The comparison between the built‐in backlash test results and the MPC CDC backlash results is presented for both Banks A and B in Figure 3.

The data of Figure 3 shows systematic offsets of differing magnitudes for Banks A and B between the MPC CDC backlash test and the built‐in backlash test. This was quantified and the mean offset for Bank A was found to be 0.08 mm ± 0.05 (2SD) and 0.04 mm ± 0.04 (2SD) for Bank B. These offsets are outside the MPC repeatability for both banks. Correlation coefficients between the two methods were calculated to be 0.84 for Bank A and 0.90 for Bank B indicating strong correlation between methods for both banks.

#### 3.2.3 Jaw positional accuracy

**FIGURE 4:**
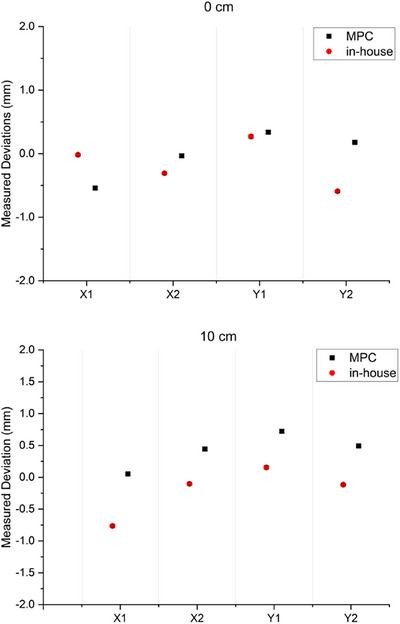
Jaw position agreement between MPC CDC and the in‐house method over a 5‐month (*n* = 5) period for the linac with the worst systematic agreement. Two jaw positions are presented; 0 and 10 cm off‐axis. Data presented as mean ± two standard deviations. Error bars are small as to be indiscernible. The y‐axis scale represents the MPC defined threshold range for the y jaws for this test.

From all jaw positioning concordance results average agreement between methods for all four jaws to within 0.4 mm at the 0 cm off‐axis position and to within 0.71 mm at the 10 cm off‐axis positions has been demonstrated. The maximum difference measured was 0.79  and 1.14 mm for the 0 and 10 cm positions, respectively. The 10 cm off‐axis position results of Figure 4 show a systematic offset between methods in the range of 0.2–0.8 mm. This is consistently observed between linacs and is not apparent in the 0 cm data for any linac.

#### 3.2.4 Jaw parallelism accuracy

**FIGURE 5:**
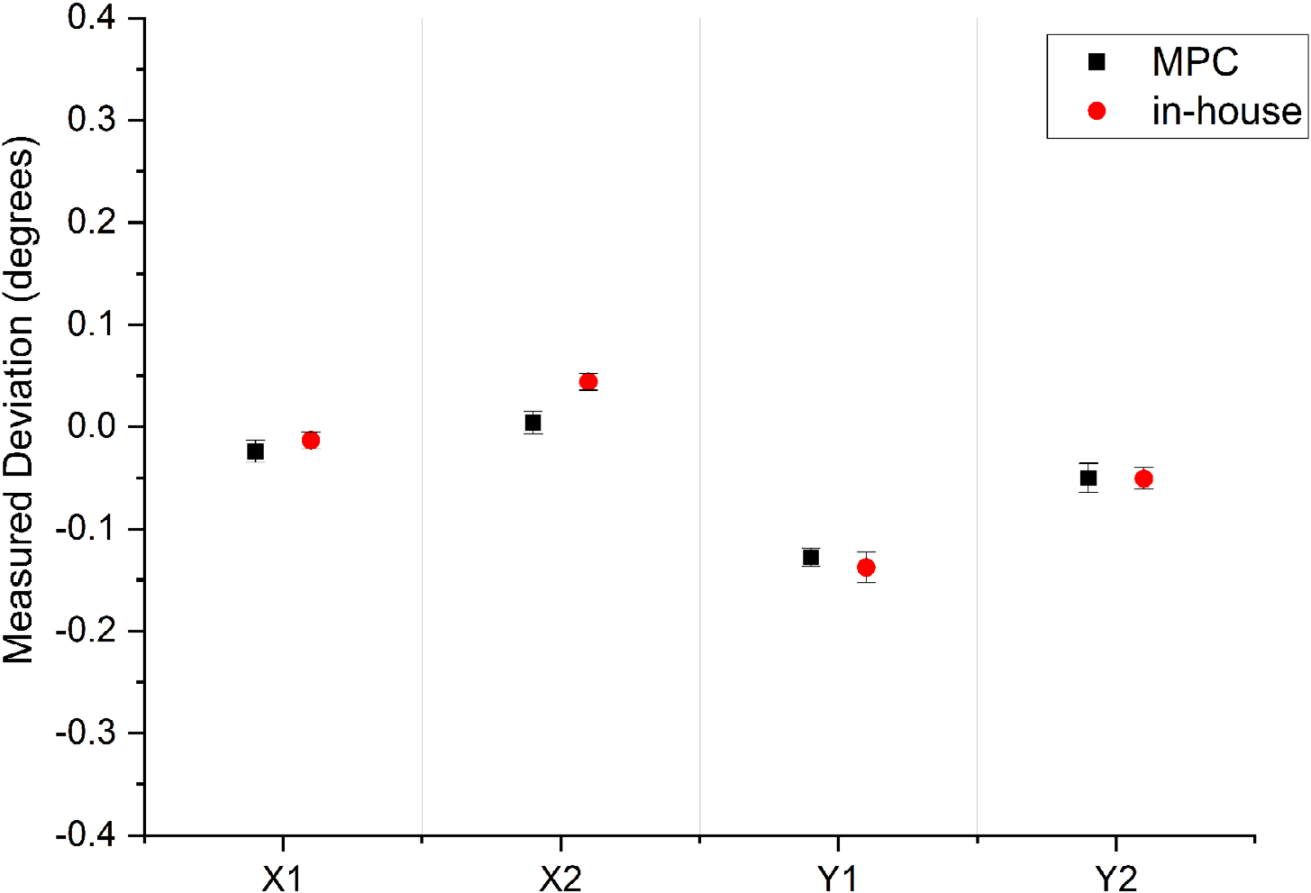
Jaw parallelism agreement between MPC CDC and the in‐house method over a 5‐month period (*n* = 5) for the linac with the largest observed measured deviations. Measured deviations presented as mean ± two standard deviations. The y‐axis scale represents the MPC defined threshold range for this test.

The results of Figure 5 show that over the 5‐month period that even for the linac reporting the greatest deviations that the MPC CDC measured jaw parallelism was consistently in agreement to in‐house EPID‐based measurements with worst agreement measured at 0.06° across all jaws for all four linacs.

### 3.3 Jaw positioning sensitivity

The results of the jaw mis‐calibration sensitivity experiment are presented in Table 1.

**TABLE 1:** MPC CDC and local department EPID‐based jaw positioning QA measured change in jaw position pre‐ and post‐jaw mis‐calibration (mm).

Jaw	MPC CDC	Local method	Difference (MPC CDC—local)
X1	−1.99	−1.82	−0.17
X2	−2.08	−1.91	−0.17
Y1	−1.09	−1.17	0.08
Y2	−1.72	−1.24	−0.48

The results of Table 1 show agreement in the measured change in jaw position between MPC CDC and local EPID method is generally within 0.17 mm except for the Y2 jaw, which is within 0.48 mm. The measured differences are within the magnitude of the backlash, which was measured to be 0.66, 0.66, 0.50, and 0.51 mm for X1, X2, Y1, and Y2 jaws, respectively, from the built‐in test.

### 3.4 MLC positioning sensitivity

The results of the MLC positioning sensitivity MLC gap adjustment experiment are presented in Table 2.

**TABLE 2:** Mean measured change in MLC position for introduced MLC gap changes for MPC CDC and the Stakitt test (mean measured change ± 2 SD).

	Measured change (mm)		
Nominal introduced change (mm)	MPC CDC	Stakitt	Difference in mean (MPC CDC—Stakitt)
Bank A			
0.25	0.34 ± 0.26	0.30 ± 0.03	0.04
0.5	0.59 ± 0.28	0.52 ± 0.04	0.07
1.0	1.08 ± 0.30	1.01 ± 0.07	0.07
Bank B			
0.25	0.34 ± 0.27	0.18 ± 0.07	0.16
0.5	0.60 ± 0.32	0.46 ± 0.06	0.14
1.0	1.12 ± 0.32	0.94 ± 0.10	0.18

The results of Table 2 show mean agreement between MPC CDC and Stakitt to within 0.18 mm for detection of a nominal 1 mm change in MLC position.

When MPC CDC is assessed for each of the three measurement positions rather than combined, results show that the nominal introduced error is measured to within 0.1 mm at two of the three positions, but up to 0.26 mm for the third position. The positions with the worst agreement were Position 2 for Bank A and position 1 for Bank B.

The results of the MLC offset adjustment experiment showed agreement between mean MPC CDC MLC position and mean Stakitt results for detecting a 0.2 mm introduced MLC offset to ‐0.08 mm for Bank A and ‐0.07 mm for Bank B. Of note was that the Stakitt mean measured changes were 0.22 mm for both banks which were in better agreement with the nominal introduced change than MPC which ranged between 0.18 and 0.3 mm.

## 4 DISCUSSION

The MPC CDC test suite has been evaluated against conventional QA methods in terms of repeatability and concordance over a 5‐month period along with sensitivity to introduced errors. For both MLC and jaws, results suggest equivalence to clinically acceptable levels between MPC CDC and the conventional QA methods used for assessment. Table 3 provides a summary of the standard QA tests for which MPC CDC could potentially be considered equivalent.

**TABLE 3:** MPC CDC tests listed against standard QA tests for which they could potentially be considered equivalent.

MPC CDC test	Equivalent standard QA test
MLC positioning	Quantitative picket fence test at Gantry 0 degrees
Jaw positioning	Standard jaw positioning test
MLC backlash	Equivalent to Varian in‐built backlash test. When combined with MLC positioning test, potential equivalence to the picket fence test at cardinal gantry angles.
Jaw parallelism	Not tested in standard QA programs.

Over a 5‐month period across four linacs including two MLC types, consistent mean agreement was observed between the MPC CDC MLC positional test and an advanced picket fence style test (Stakitt). Worse agreement was observed for MLC Bank A compared to Bank B with a maximum 0.32 mm mean difference observed between MPC CDC and Stakitt. The magnitude of this offset is at about 65% of the ± 0.5 mm tolerance of AAPM MPPG 8.b and hence is considered clinically acceptable.

The two standard deviation variability in Bank B MPC concordance measurements was as high as ± 0.49 mm. This is of the order of the AAPM MPPG8.b. proposed tolerance level. However, this result is an outlier and was only observed in one bank on one linac with the spread in all other results at approximately half of this magnitude.

The MLC sensitivity experiment results show that the mean measured change in MLC position was in agreement between MPC and Stakitt to within 0.18 mm for a nominal 1 mm change. This result would suggest comparable sensitivity between methods in the context of the 0.5 mm proposed tolerance. Better agreement was observed between Stakitt and MPC for Bank A rather than Bank B. This is opposite to the concordance results of Figure 2. However, both are hypothesized to be due to the influence of backlash following the reasoning of Barnes et al.^19^ supplementary material. In the MPC CDC delivery, MLC leaves are always pushed into their measurement position whereas with the Stakitt test one bank is pushed into position and the other pulled into position as the leaves move across the field. As presented in Barnes et al.,^19^ in such circumstances, one leaf bank will have no backlash error introduced into the leaf positioning while the other bank will have the whole of the backlash translated into leaf positioning error. The 0.25 mm magnitude of the difference between MPC CDC and Stakitt for Bank A is consistent with the expected backlash as presented in Figure 3 and hence supports the hypothesis.

In this study, a strong correlation was observed between the MPC backlash results and the Varian built‐in backlash test albeit with a systematic offset observed between the two methods. This offset is likely due to differences in the respective methodology, which Varian account for in the respective tests different tolerances. The MPC CDC backlash threshold of 0.5 mm is 0.1 mm larger than the built‐in backlash test tolerance. This difference approximately equates to the 0.08 mm offset between methods observed. Taking these differences in tolerance into account for the measured systematic offset between methods, the results indicate that the two methods can be considered equivalent.

The maximum differences in measured jaw positions between methods were recorded for upper Y jaws. In MPC, the upper (Y) jaw threshold is set at 2 mm, while the lower (X) jaw is set to 1 mm. Thresholds are set this way because for the same mechanical offset in the jaw position the upper jaw will project a greater offset at isocenter height than the lower jaw. In the context of the 2 mm threshold for the Y jaw, the maximum difference recorded between methods of 1.14 mm could still be considered acceptable. In terms of sensitivity to jaw error, the results of Table 1 show agreement to within 0.17 mm for the X1, X2, and Y1 jaws and this is considered to show excellent equivalence in the clinical context. The agreement of up to 0.48 mm for the Y2 jaw is considered to show clinically acceptable equivalence considering the magnitude of the jaw backlash and the increased MPC tolerance for the upper (Y) jaws of ± 2 mm. It is unclear why the Y2 jaw is an outlier in the sensitivity experiment, but it is noted that results are within the measured backlash. The agreement of MPC CDC and the in‐house method for assessing jaw parallelism of 0.1° is much less than the 0.4° tolerance of the MPC CDC and hence considered clinically acceptable.

The systematic offsets in measured jaw position observed between methods in Figure 4 are hypothesized to be due to differences in the methods. Differences in jaw motions prior to moving into the final measurement position would affect the influence of backlash in terms of whether the backlash translates into positional error or not. If jaw backlash translates fully into jaw positional error as it does for the MLC as presented in Barnes et al.,^19^ then the backlash becomes an uncertainty in the measurement. It is noted that the results show that the 10 cm off‐axis agreement to be worse than the 0 cm agreement. It is uncertain why this is, but could be due to backlash because the jaws were moved into position from different directions.

As an MLC positional test, the MPC CDC has a number of positive attributes for use in instances for which the picket fence test would standardly be used. The CDC assesses individual MLC leaf positions at multiple points along their travel spatially referenced to the collimator rotation axis. These attributes are those of an advanced quantitative picket fence style test.^20^ The measured CDC repeatability, accuracy, and sensitivity as presented in Figure 2 and Table 2 in comparison to such an advanced picket fence style test suggest equivalence to within clinically acceptable levels. The approximate 4‐min acquisition and analysis time make CDC an attractive option for post MLC QA when time is tight and patient schedules have been delayed. As such, the results of this study suggest that the MPC CDC MLC tests could safely be used in place of a picket fence style test and would be a quick and convenient means of doing so. A potential pathway forward is the Hybrid QA approach, as espoused by Pearson et al.,^9^ whereby the MPC CDC be utilized for routine picket fence style MLC QA and return to clinical use testing. MPC CDC would be complemented with conventional QA methods, performed at regular intervals, with analysis of the concordance between the two methods used as an independent ongoing verification of the MPC CDC performance.

In comparison to the standard MPC jaw positional test, the CDC test checks additional points along the jaw travel, which is considered to be an advantage. The MPC jaw parallelism test is considered to be equivalent to conventional QA based upon the results of this study. Although such parallelism is not expected to change, its verification is considered useful.

Acknowledged weaknesses of this study for the evaluation of the CDC MLC positioning include that the CDC MLC positions compared to the Stakitt test are not exactly the same, but are closest fit. Differences in measurement positions between Stakitt and MPC CDC also mean that CDC MLC Pos 1 and Pos 2 positions have not been assessed. Also, the commonality of EPID as the detector for both MPC CDC and comparison methods is not ideal. However, inaccuracies in other potential detectors, particularly with spatial resolution and accuracy of setting the collimator‐axis reference point mean that alternate detectors likely do not have the required accuracy to provide a meaningful comparison to MPC CDC. If such methods are or become available, then they are recommended to be performed as an extension to this study.

In terms of jaw testing weaknesses, the CDC test retains the issue of standard MPC in being susceptible to focal spot mis‐alignment due to the MLC being used to determine the spatial reference point as presented in Barnes et al.^3^ and Chojnowski et al.^23^ Also, with the jaw backlash measured to be of the order of 0.5 mm, the uncertainties in jaw positioning mean that jaw positional accuracy to the order of tenths of a millimeter is currently not feasible.

For strict compliance with AAPM best practice recommendations,^14^, ^15^, ^16^ the CDC would ideally require functionality to be performed at non‐zero cardinal gantry angles. This is something that Varian could consider for future versions of MPC CDC. The studies of Barnes et al.^19^ and Barnes et al.^20^ suggest that the requirement for non‐zero gantry angle MLC positional testing is primarily due to the impact of MLC backlash on MLC positioning when gravity is in the direction of MLC motion. The MPC CDC includes a measure of MLC backlash for which the results of this study suggest to be equivalent to the Varian built‐in backlash test. Considering this, the current MPC CDC including Gantry 0 positional measurement couple with backlash assessment could be considered to provide equivalent MLC testing to the picket fence test measured at each cardinal gantry angle. The MLC sensitivity results indicate MPC CDC equivalence within clinically significant levels to an advanced picket fence style test. Considering that the advanced picket fence style test has demonstrated accuracy to allow for a ± 0.5 mm tolerance at gantry zero^20^ suggest that such a tolerance is also appropriate for MPC CDC. However, since MPC tolerances are fixed and in the case of MLC positional accuracy are set at 1 mm then either a local tolerance would need to be applied or Varian would need to adjust the tolerance in MPC or make it user customizable. Altogether, this study indicates that the MPC CDC MLC positional test could be considered to meet the requirements of AAPM best practice recommendations of AAPM MPPG 8.b.

## 5 CONCLUSION

MPC CDC tests have been demonstrated to provide equivalent testing of the MLC and jaws with standard methods to within clinically acceptable levels. In combination with the MPC CDC MLC backlash test, which has also demonstrated equivalence to the conventional test method, the MPC CDC could potentially be considered to provide equivalent MLC testing to the picket fence test at cardinal gantry angles. Hence, the MPC CDC MLC test is considered capable of meeting the requirements of AAPM best practice MLC QA including that of AAPM MPPG 8.b. Since MPC CDC has been shown to be both quick and convenient to perform and as it comes standardly with the TrueBeam linac, it is considered a useful means of assuring MLC positional accuracy, especially in low‐resourced departments.

## AUTHOR CONTRIBUTIONS

Michael Barnes: Had the original idea for the study, scoped the project and determined experimental methodology, organized data acquisition, analyzed the repeatability, MLC backlash concordance and all sensitivity experiment and primarily wrote the manuscript. Andrew Dipuglia: Developed methodology and performed analysis for the jaw parallelism concordance assessment, analyzed the jaw position concordance data and provided scientific input into the manuscript. Brad Beeksma: Analyzed the MLC concordance data and provided scientific input into the manuscript. Joerg Lehmann: Provided review of the manuscript prior to submission and scientific input into its development.

## CONFLICT OF INTEREST STATEMENT

The authors acknowledge that they have no conflicts of interest relevant to this study.

## Data Availability

Data is not made available for this study.
